# The Effects of Adult Ageing and Culture on the Tower of London Task

**DOI:** 10.3389/fpsyg.2021.631458

**Published:** 2021-02-22

**Authors:** Louise H. Phillips, Louisa Lawrie, Alexandre Schaefer, Cher Yi Tan, Min Hooi Yong

**Affiliations:** ^1^School of Psychology, University of Aberdeen, Aberdeen, United Kingdom; ^2^Department of Psychology, Monash University Malaysia, Sunway City, Malaysia; ^3^Department of Psychology, Sunway University, Sunway City, Malaysia; ^4^Aging Health and Well-being Research Centre, Sunway University, Sunway City, Malaysia

**Keywords:** planning, ageing, culture, working memory, Western, Asian

## Abstract

Planning ability is important in everyday functioning, and a key measure to assess the preparation and execution of plans is the Tower of London (ToL) task. Previous studies indicate that older adults are often less accurate than the young on the ToL and that there may be cultural differences in performance on the task. However, potential interactions between age and culture have not previously been explored. In the current study we examined the effects of age on ToL performance in an Asian culture (Malaysia) and a Western culture (British) (*n* = 191). We also explored whether working memory, age, education, and socioeconomic status explained variance in ToL performance across these two cultures. Results indicated that age effects on ToL performance were greater in the Malaysian sample. Subsequent moderated mediation analysis revealed differences between the two cultures (British vs Malaysians), in that the age-related variance in ToL accuracy was accounted for by WM capacity at low and medium education levels only in the Malaysian sample. Demographic variables could not explain additional variance in ToL speed or accuracy. These results may reflect cultural differences in the familiarity and cognitive load of carrying out complex planning tasks.

## Introduction

With advancing age, older adults often experience a range of cognitive deficits across multiple domains, including executive function (EF) (Rey-Mermet and Gade, 2018; Jaroslawska and Rhodes, 2019). EF is often categorised as a set of high-level cognitive processes important for self-regulation and adaptation to complex tasks. EF is supported mainly by the prefrontal cortex and it is operationalized by a variety of tasks such as tasks involving memory updating, shifting and cognitive inhibition (Friedman and Miyake, 2017). Planning is often considered an important skill within the EF domain because it is crucial for everyday tasks such as shopping and cooking, as well as for many other work-based tasks (McGeorge et al., 2001). Successful planning involves making an initial ordering of steps to reach a goal, task execution, monitoring progress and dealing with unexpected events.

The tower tasks - Tower of Hanoi “ToH,” and Tower of London “ToL” – introduced by Simon (1975) and Shallice (1982) are frequently used to assess an individual’s planning ability. They are particularly useful in assessing EF for patients with brain damage, particularly in the frontal lobes (Sullivan et al., 2009; Szczepanski and Knight, 2014). Disturbance of frontal lobe networks is often associated with poor inhibition and working memory (WM) deficits, which then may affect planning ability. Compared to controls, individuals with lateral prefrontal cortex damage were found to be slower and required more moves in these Tower planning tasks (Shallice, 1982; Owen et al., 1990; Goel and Grafman, 1995).

In the ToL task (Shallice, 1982), participants are required to move three balls from a starting position to a predetermined goal state in a minimum number of moves. Successful performance on the task would require a sequence of moves planned in WM, executed, monitored, and revised prior to making an action. As suggested by Berg and Byrd (2002), ToL performance reflects a combination of both planning and visuo-spatial problem solving. They suggested four possible measures of performance; (1) accuracy (how many problems are solved), (2) efficiency (number of moves needed to solve the problems over the minimum possible), (3) reaction time (RT)/speed (how fast a participant could complete one or all problems), and (4) rule breaks. The perfect scorer would solve all problems without breaking a rule, using the allowed number of moves for the problems and in the shortest time possible. Agranovich et al. (2011) reported that Russian participants were slower but more accurate performance in the ToL task compared to US counterparts, suggesting that assessing both speed and accuracy may be important in the task.

Making a mental plan and then remembering, executing and adapting the plan are all tasks which are likely to depend on the capacity to update WM. Evidence indicates that WM is often associated with ToL (Welsh et al., 1999; Rönnlund et al., 2001; Gilhooly et al., 2002; Asato et al., 2006; D’Antuono et al., 2017). Phillips (1999) reported that having good verbal and visuospatial memory had a direct relationship to ToL success, which further highlights the importance of these functions during planning. However, other studies have reported that fluid intelligence, and not WM contributed to ToL success (Unterrainer et al., 2004; Zook et al., 2006). However, Zook et al. (2006) observed that WM was a contributor to the ToH task, implying that a more complex task such as ToH imposes a heavier cognitive load on WM.

Other factors such as age, years of education, and socioeconomic-status (SES) have been reported to contribute towards ToL success but this is rather inconsistent across various studies. Age effects on ToL success has been well-documented in many studies showing young adults were generally more accurate in solving ToL trials in minimum moves compared to older adults (Bugg et al., 2006; Zook et al., 2006; Boccia et al., 2017; D’Antuono et al., 2017), with the exception of two studies (Gilhooly et al., 1999; Phillips et al., 2003). ToL performance has been observed to be progressively worse with increasing age among samples of older people (Köstering et al., 2014; D’Antuono et al., 2017; Unterrainer et al., 2019). Some studies have reported fewer errors of planning (Gilhooly et al., 1999), and faster planning and execution times in young adults (Phillips et al., 2003) compared to older adults, which again demonstrated age differences in the task. Some studies reported that higher education (measured by years of schooling) is positively correlated to ToL performance (Boccia et al., 2017; D’Antuono et al., 2017; Unterrainer et al., 2019). This is not surprising as many other studies have demonstrated a relationship between education and EF performance, and education may act as a protective factor against cognitive ageing. SES is also considered an important factor as the living environment in both childhood and adulthood has a long term impact on cognition (Hanscombe et al., 2012; Lyu and Burr, 2016). There is evidence that children from high SES families exhibited better cognitive control compared to low SES families (Morton and Harper, 2007), and higher accuracy in ToL tasks (Malloy-Diniz et al., 2008; Naeem et al., 2018).

However, all of these studies have been carried out in Western samples, and very little is known about the influence of age, education and SES on planning performance in people from other cultures. Further, the evidence on ageing effects on ToL performance has only been reported in Western populations, and we do not know whether similar age effects are applicable in an Asian ageing sample. To best of our knowledge, we found only two studies that compared Asian and Western samples on a ToH task, and both focused on children (Ellefson et al., 2017; Xu et al., 2020). In these studies performance on the ToH task was considered as one component in a broader EF score. In the two cross-cultural comparison studies, children from China and Hong Kong were more accurate and faster in EF tasks compared to United Kingdom age-matched sample (Ellefson et al., 2017; Xu et al., 2020). However there were no cultural differences in EF performance for the parents (Ellefson et al., 2017), suggesting that there may be generational or developmental differences in the effects of culture on EFs. Some postulated that the difference in EF performance was partly driven by cultural differences in educational and parenting practices (Sabbagh et al., 2006; Ellefson et al., 2017). It remains to be seen whether the cultural differences reported in these studies in childhood can be seen in older adults.

It is therefore of interest how effects of age and culture may interact to influence performance in a planning task. In an influential paper Park et al. (1999) proposed that the distinction between cognitive hardware or “mechanics” and acquired cultural knowledge or “pragmatics” becomes more pronounced with age. Specifically, tasks which are most dependent on fundamental cognitive processes should show similar age effects in different cultures. Supporting this, some studies indicate very similar age effects on speed and memory in Western and Eastern cultures (Park et al., 1999; Chua et al., 2006; Gutchess et al., 2006). In contrast, tasks which are more culturally loaded should show a greater divergence of age-related trajectories across different cultures. Indeed, there is evidence that the influence of age on some aspects of WM (Hedden et al., 2002) and theory of mind (Phillips et al., 2020) is greater in Asian compared to Western samples. It has been argued that this may occur because these tasks involve processing styles which are not the cultural norm in Asian societies, and while younger adults have enough cognitive capacity to overcome this older adults do not (Na et al., 2017). Also, this pattern of results may reflect generational changes in the familiarity of certain tasks, along with cultural differences in the pace of cognitive ageing.

In the current study we looked at the effects of age on ToL performance in a Western (British) and Asian (Malaysian) culture, while taking into account individual differences in WM, education and subjective SES. We used a physical version of ToL blocks and pegs to minimise the technological barriers sometimes experienced among older adults (Heart and Kalderon, 2013). Given previous findings of greater age differences in WM capacity in Asian compared to Western samples (Hedden et al., 2002), and the known involvement of WM in the planning processes involved in ToL tasks (Gilhooly et al., 2002), we made the following predictions: (1) There will be an interaction between age and culture reflecting a greater detrimental effect of old age for Malaysians compared to the British participants. (2) The age effect will be mediated by age differences in WM, and this may be moderated by culture, so that the mediating effects of WM are greater in the Malaysian sample. The main measure from the ToL was the accuracy of solving the trials, and we also looked at the time taken to index the efficiency of processing: separated into planning and execution times.

## Materials and Methods

### Participants

A total of 191 participants completed the ToL task in this study. It was a follow-up to another larger study (Phillips et al., 2020), so the participants described here are a subset of those reported in that study. Participants were recruited from two locations; United Kingdom and Malaysia. The Malaysian sample was recruited from the capital city, Kuala Lumpur and surrounding areas while the United Kingdom sample was recruited from northeast Scotland (Aberdeen and Aberdeenshire). They were recruited from university bulletin services, course credit completion, local senior citizen social community clubs, local participant panels, and references from other participants. All participants had normal or corrected-to-normal vision, not colour-blind, self-reported as being currently healthy, and were living independently in the community. None of the participants reported having any neurological illnesses or previous brain injuries (see Table 1 for details). All participants gave their written informed consent for inclusion in the study before participating. The study protocol was approved by respective research ethics committee in both institutions.

**TABLE 1:** Participants demographic information with means and standard deviations in brackets (*n* = 191).

	**British**		**Malaysian**				
	**Young (*n* = 33)**	**Old (*n* = 40)**	**Young (*n* = 73)**	**Old (*n* = 45)**	**Main effect of age**	**Main effect of culture**	**Interaction of age × culture**
Gender	M: 3 F: 30	M: 14 F: 26	M: 27 F: 46	M: 19 F: 26	−		
Mean Age	21.85 (2.33)	68.37 (6.15)	20.68 (1.83)	70.33 (6.55)	−		
Age Range	18-28	60-89	18-30	60-88	−		
Years of Education	15.61 (1.82)	15.72 (3.32)	12.44 (0.97)	13.78 (2.65)	4.71*	58.32***	3.36
Subjective SES^*a*^	6.11 (1.87)	7.01 (1.30)	5.23 (1.35)	5.76 (1.37)	10.64**	23.63***	0.77
HADS^*b*^-Anxiety	9.45 (5.20)	4.97 (3.29)	9.60 (3.76)	5.80 (2.90)	51.55***	0.71	0.35
HADS^*b*^-Depression	4.91 (3.81)	3.36 (2.60)	5.01 (2.92)	4.31 (2.64)	6.13*	1.34	0.86
MoCA^*c*^	n/a	28.30^†^ (1.10)	n/a	26.18 (2.47)	−	3.91***	

^*a*^Socio-economic status. ^*b*^Hospital Anxiety and Depression Scale. ^*c*^Montreal Cognitive Assessment using *t* test. ^†^23 adults only. *p < 0.05, **p < 0.01, and ***p < 0.001.

### Stimuli/Procedure

All participants were tested in laboratories either in Scotland or Malaysia. They completed the questionnaires on paper, *n*-back task on the computer, and the physical ToL task.

Participants completed a short demographics questionnaire, the self-reported MacArthur Ladder Scale as a measure of subjective levels of SES (Adler et al., 2008), the Montreal Cognitive Assessment (MoCA) for older adults only (Nasreddine et al., 2005) and the Hospital Anxiety and Depression Scale (HADS) (Zigmond and Snaith, 1983). Questionnaires were completed before moving onto the ToL and a WM task (outlined below) in a counterbalanced order. Other tasks and questionnaires not considered here were also completed.

The MacArthur Ladder refers to how one perceives themselves relative to others in their own community (Adler et al., 2008). Each participant rated themselves on a ladder with ten rungs positioned vertically with the following instruction. “Imagine that this ladder pictures how the society in Malaysia is set up. At the top of the ladder are the people who have the most money, most education, and most respected jobs. At the bottom are the people who have the least money, least education, and least respected jobs or no job. The higher up you are on this ladder, the closer you are to the people at the very top, and the lower you are, the closer you are to the people at the very bottom. Where would you place yourself on this ladder? Please place an “X” on the rung where you think you stand at this time in your life, relative to others.” They were informed to mark a cross on a specific rung, and that rung corresponds to a number between 1 (very low) to 10 (very high).

For this study, we chose to include subjective SES instead of the objective SES to ensure measurement consistency across countries. For example, it is possible to identify SES by postcodes in the United Kingdom but this is not available in Malaysia. Income inequality and country affluence are also reported differently in each country. Further, objective SES measures such as income, education and occupation tend to overlook other factors such as age, ethnicity, indigeneity, and rurality (Rubin et al., 2014). The subjective definitions of social class and SES helps shape how an individual perceives themselves in a community and a nation, and thus places a different emphasis on SES.

### Tower of London (ToL) Task

The ToL task required the movement of three different-coloured wooden balls across three wooden pegs of different length to duplicate the goal state within a designated number of moves. A set of instructions were read out to the participants and we verbally sought affirmation before continuing. Participants were video-recorded throughout the task to measure total time taken in completing the problems.

Using printed coloured pictures, participants were told to transform the start state to a goal state in the least number of moves possible while following the rules where: (1) only one ball may be moved at a time; (2) no ball may be held or placed outside the pegs while another ball is being moved; (3) one ball can be placed on the shortest peg, two balls on the medium peg and three balls on the tallest peg. Our ToL trials were based on Shallice’s (1982) study consisting of twelve problem sets with increasing difficulty. Participants were told to plan their moves prior to beginning each trial, and were allowed three attempts for each problem. We followed the scoring procedures proposed by Krikorian et al. (1994) in which, for each trial, three points were given for a successful solution at first attempt (i.e., within the allowed number of moves); two points for successful solution at second attempt, one point for successful solution at third attempt and no points were given if participants failed to complete the task within three attempts. A total score was obtained from the sum of points earned on all twelve trials (maximum possible = 36).

We also included measures of time taken to plan “ToL planning” and time to execute “ToL execution” each problem. For plan time, we recorded the time from the instruction “Now, make it look like this….” to the first ball placement. For execution time, time taken was measured from the first ball placement to the point when they released their hand from the last ball. All time measurements were recorded in seconds.

### Working Memory

Working memory was assessed with a 2-back task (Braver et al., 1997; Jaeggi et al., 2010). Before starting the 2-back task, they completed 10 practice trials of a 1-back task. A series of digits from 1 to 9 were sequentially presented on the centre of a screen. Participants had to respond to each digit with a keypress to indicate whether it matched the digit presented immediately before. Each digit was presented on screen for at least 1,300 ms or until participants responded, and the order of presentation of the digits was randomized between participants. Then they received instructions for the 2-back task. Participants were told to respond when the third digit was presented on-screen, and to indicate by a keypress whether the digit matched the identity of the digit presented two positions back in the sequence. Thereafter, participants made continuous responses for each digit presented. Participants completed eight practice trials of the 2-back before commencing the main experimental block, which was composed of 78 trials, 20 of which were “target” trials (i.e., trials for which the digit actually matches the digit presented 2 positions back). The dependent variable was percent accuracy in identifying target trials and non-target trials correctly in the 2-back only.

### Data Analysis

We checked for normality and outliers in our data. There were no outliers in the ToL accuracy and total time, and WM (applying 3SD above and below mean). Initial analyses then investigated whether there were effects of age and culture on each of our demographic variables (years of education, SES, HADS anxiety, and depression). We conducted analysis of covariance (ANCOVA) looking at the effects of age group (young, old) and culture (British, Malaysian) for our dependent variables in the ToL task (accuracy, planning, and execution time taken). To ensure that any effects were not due to sociodemographic factors, we included as covariates for those shown to be affected by culture. We also completed follow up comparisons for significant age × culture interactions identified from the ANCOVA.

Following the ANCOVA, we wanted to examine the relationships between age, WM and ToL performance, and how they might be moderated by culture and education. We performed a moderated mediation analysis using age as a predictor, WM as mediator, culture and education as moderators, and ToL as the outcome variable (ToL accuracy, planning time, and execution time). This meant that we used the variables – age, WM, culture, education, ToL accuracy – together for one moderated mediation analysis, and repeat this for ToL planning and execution times. All were continuous variables except culture which was dichotomous. The moderated mediation analysis (model 75) was conducted using the PROCESS (v 3.4) macro by Hayes (2017) in SPSS version 25 using a percentile bootstrap confidence interval (CI) which was generated using 5,000 samples. If the upper and lower boundaries of the CI for a relationship contained a zero, this is considered as a non-significant result. As this model does not explicitly include the index of moderated mediation, the conditional indirect effects (refers to the moderated mediated relationship) were examined in order to further probe the moderated mediation effect.

## Results

### Age and Culture Effects on Socio Demographic Variables

To determine the effects of age and culture, we ran a series of 2 (Age group: young vs old) × 2 (culture: British vs Malaysian) analysis of variance (ANOVA) with each socio-demographic variable (years of education, SES, HADS anxiety, and depression) as the dependent variable. For descriptive and inferential statistics see Table 1.

We found main effects of age on years of education, SES, HADS – Anxiety, and HADS – Depression, all *p*s < 0.031. Results showed that young adults had fewer years of education, rated themselves as having lower SES, higher anxiety and depression compared to older adults. As for culture, we found a main effect of culture in years of education and SES, both *p*s < 0.001. Results showed that our British sample had more years of education and also rated higher on SES compared to Malaysians. There was no significant main effect of culture in Anxiety and Depression scores, both *p*s > 0.356. We did not find any significant age x culture interaction in all four variables; years of education, SES, anxiety and depression, all *p*s > 0.068.

### ToL Accuracy

Given that our analyses on sociodemographic variables unveiled a significant main effect of Culture for education and SES, we decided to include these variables as covariates in subsequent ANCOVAs.

We performed a 2 (Age group: young vs old) × 2 (British vs Malaysian) ANCOVA with ToL accuracy total scores as the dependent variable. We found that the main effect of age was significant, *F* (1,184) = 24.684, *p* < 0.001, *n*_*p*_^2^ = 0.118, with young participants having higher ToL accuracy compared to older adults. The main effect of culture was not significant, *F* (1,184) = 1.255, *p* = 0.264, *n*_*p*_^2^ = 0.007. Consistent with our hypotheses, we found a significant age by culture interaction, *F* (1,184) = 4.888, *p* = 0.028, *n*_*p*_^2^ = 0.026. None of the covariates were significant, both *p*s > 0.341.

**FIGURE 1:**
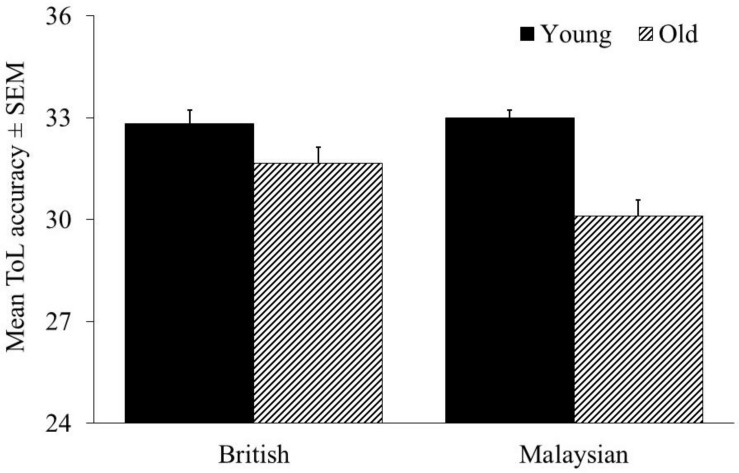
ToL accuracy for the British and Malaysian participants.

This interaction was driven by a larger effect of age for the Malaysian compared to the British sample (see Figure 1), as *post hoc* tests showed that Malaysian older adults had a significantly lower ToL accuracy compared to young adults, *t* = 5.91, *p* < 0.001, *d* = 1.06, whereas only a marginal effect of age was found in the British sample, *t* = 1.81, *p* = 0.075, *d* = 0.43.

### ToL Planning and Execution Time

**FIGURE 2:**
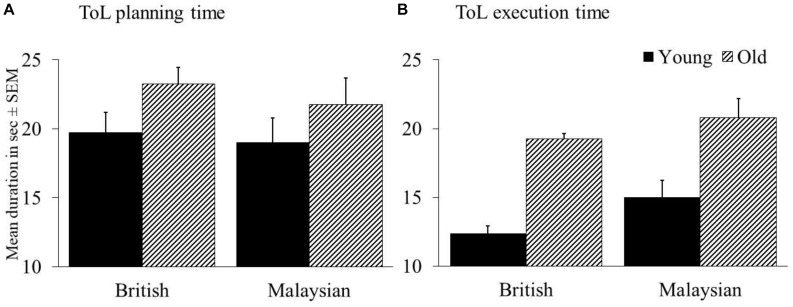
ToL planning time **(A)** and ToL execution time **(B)** for the British and Malaysian participants.

Similar to the above analyses, we completed a 2 × 2 ANCOVA with ToL planning time for all 12 problems as a dependent variable. Here, we found no significant main effect of age, *F* (1, 184) = 2.322, *p* = 0.129, *n*_*p*_^2^ = 0.012 and no significant main effect of culture, *F* (1, 184) = 0.436, *p* = 0.51, *n*_*p*_^2^ = 0.002. There was no significant interaction, *F* (1, 184) = 0.421, *p* = 0.517, *n*_*p*_^2^ = 0.002 (see Figure 2A). Education was a significant covariate, *F* (1, 184) = 8.006, *p* = 0.005, *n*_*p*_^2^ = 0.042 suggesting that education had an overall effect on ToL planning time. The other covariate was not significant, *p* = 0.657 (see Figure 1).

For execution time, we found a main effect of age, *F* (1, 184) = 41.609, *p* < 0.001, *n*_*p*_^2^ = 0.184, with young adults being much faster in executing a move compared to older adults. There was also a main effect of culture, *F* (1, 184) = 5.207, *p* = 0.024, *n*_*p*_^2^ = 0.028, showing that Malaysians were slower than the British to execute a move. However, there was no significant interaction of age × culture, *F* (1, 184) = 0.497, *p* = 0.482, *n*_*p*_^2^ = 0.003 (see Figure 2B). Both covariates were not significant, *p*s > 0.526. *Post hoc* tests results showed that both British, *t* = 4.867, *p* < 0.001, *d* = 1.19 and Malaysian older adults *t* = 4.918, *p* < 0.001, *d* = 0.84 took a longer time in executing a move compared to young adults.

### Working Memory

**FIGURE 3:**
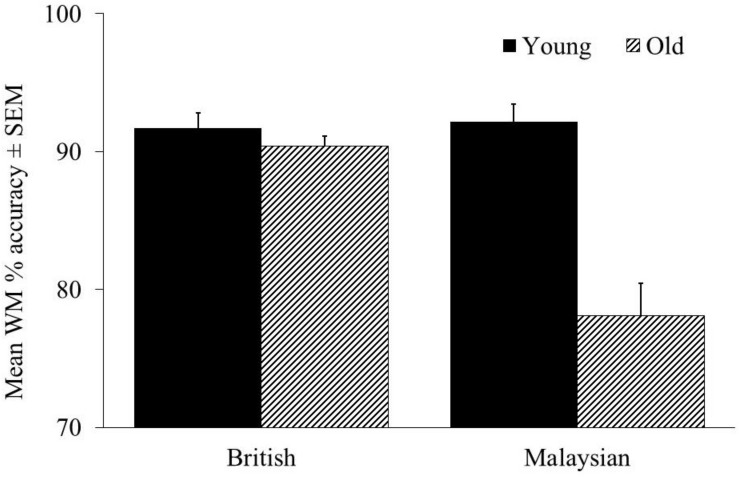
WM accuracy for the British and Malaysian participants. WM, working memory; E, education; C, culture is coded as 1 = British and 2 = Malaysian. *p < 0.05, **p < 0.01, and ***p < 0.001.

A 2 (age group) × 2 (culture) ANCOVA on WM accuracy was conducted. We found a main effect of age, *F* (1, 184) = 36.045, *p* < 0.001, *n*_*p*_^2^ = 0.164, whereas culture was not significant, *F* (1, 184) = 2.531, *p* = 0.113, *n*_*p*_^2^ = 0.014. Younger adults were more accurate in WM compared to older adults (see Figure 3). There was a significant interaction between age and culture, *F* (1, 184) = 22.827, *p* < 0.001, *n*_*p*_^2^ = 0.110. Here, we found that education was a significant covariate, *F* (1, 184) = 11.510, *p* < 0.001, *n*_*p*_^2^ = 0.059 but not for SES, *F* (1, 184) = 0.797, *p* = 0.373, *n*_*p*_^2^ = 0.004. *Post hoc* tests showed that Malaysian older adults performed worse than young adults, *t* = 6.80, *p* < 0.001, *d* = 1.17, but this was not the case for the British sample, *t* = 0.767, *p* = 0.445, *d* = 0.18 (See Figure 3).

### Moderated Mediation Analyses

In our earlier analyses, we found that older adults had lower ToL accuracy, slower in executing a move and also had poorer WM accuracy compared to young adults. The correlations analysis showed that age was positively correlated with planning time (*r* = 0.155, *p* = 0.032) and ToL execution time (*r* = 0.458, *p* < 0.001) and negatively correlated to WM (*r* = −0.388, *p* < 0.001) and ToL accuracy (*r* = −0.393, *p* < 0.001) (see Table 2). From the earlier ANCOVA analyses, results showed that education was a significant covariate in ToL planning and WM and that SES was not significant in any of our dependent variables. Because of this, we included education as a moderator and considered that culture may play a role in the relationship between age and WM. We conducted a moderated mediation analysis (model 75) on SPSS version 25 with age as a predictor, WM as mediator, culture and education as moderators to path a “age and WM,” and path b “WM and dependent variable,” and ToL as dependent variable (ToL accuracy, planning, and execution time) (see Table 3 for details). As education is a continuous variable, it was mean-centered prior to the mediation analysis. The values of education at which we probed the indirect effect referred to three centerings: low (mean − 1SD = −2.617), medium (mean = 0.00) and high levels of education (mean + 1SD = 2.617).

**TABLE 2:** Means, standard deviations and correlations for study variables in the whole sample (*n* = 191).

**Variable**	***M***	***SD***	**1**	**2**	**3**	**4**	**5**	**6**
1. Age	42.570	24.520	−					
2. Culture	−	−	−0.153*	−				
3. Education	13.988	2.617	0.241**	−0.506***	−			
4. Working memory	88.415	11.230	−0.388***	−0.182*	0.186*	−		
5. ToL accuracy	31.990	2.881	−0.393***	−0.050	0.001	0.445***	−	
6. ToL planning time	20.672	10.981	0.155*	−0.071	0.223**	0.047	0.077	−
7. ToL execution time	16.824	6.838	0.458***	0.077	0.058	−0.324***	−0.561***	0.261***

*p < 0.05, **p < 0.01, and ***p < 0.001. Culture is coded as 1 = British and 2 = Malaysia. ToL, Tower of London.

**TABLE 3:** Regression coefficients for moderated mediation model.

**Independent variable**	**Dependent variable**	**Standardised coefficient**	**SE**	***t***	***p***	**95% CI (lower)**	**95% CI (upper)**
Age	WM	**0.4469**	0.0803	2.5481	0.0116	0.0462	0.3632
Age	Tol Accuracy	**−0.2783**	0.0092	−3.5608	0.0005	−0.0508	−0.0146
Age	Tol Planning Time	0.1581	0.0367	1.9303	0.0551	−0.0016	0.1431
Age	Tol Execution Time	**0.4314**	0.0190	6.3426	<0.001	0.0829	0.1577
WM	Tol Accuracy	**1.1651**	0.0919	0.2512	0.0014	0.1175	0.4803
WM	Tol Planning Time	0.1245	0.3715	0.3276	0.7436	−0.6113	0.8547
WM	Tol Execution Time	−**0.9575**	0.2265	−2.5743	0.0108	−1.0298	−0.1362
C	WM	−0.1266	1.5248	−1.9146	0.0571	−5.9277	0.0889
C	Tol Accuracy	−0.0210	0.4560	-0.2719	0.7860	−1.0236	0.7756
C	Tol Planning Time	0.0451	1.8757	0.5421	0.5884	−2.6839	4.7174
C	Tol Execution Time	0.1059	0.8593	1.7309	0.0852	−0.2080	3.1827
E	WM	**0.2208**	0.3162	2.9964	0.0031	0.3236	1.5712
E	Tol Accuracy	−0.0462	0.0961	−0.5297	0.5970	−0.2406	0.1387
E	Tol Planning Time	**0.1897**	0.3400	2.3414	0.0203	0.1253	1.4667
E	Tol Execution Time	0.0741	0.3143	0.6164	0.5384	−0.4263	0.8137
Age × C	WM	−**0.2710**	0.0569	-4.4766	<0.001	−0.3672	−0.1426
Age × E	WM	0.0754	0.0158	0.8391	0.4025	0.0179	0.0444
C × WM	Tol Accuracy	−**0.2225**	0.0529	−2.2150	0.0280	−0.2215	−0.0128
C × WM	Tol Planning Time	−0.0275	0.2329	−0.2370	0.8130	−0.5146	0.4042
C × WM	Tol Execution Time	**0.2063**	0.1145	2.2534	0.0254	0.0321	0.4839
E X WM	Tol Accuracy	−0.0194	0.0084	−0.2246	0.8225	-0.0185	0.0147
E × WM	Tol Planning Time	−0.1293	0.0417	−1.1593	0.2478	−0.1306	0.0339
E × WM	Tol Execution Time	−0.0739	0.0289	−0.5957	0.5521	−0.0741	0.0398

*WM, working memory; ToL, Tower of London; SE, standard error; CI, confidence interval. The bolded values meant that the finding was significant.*

**FIGURE 4:**
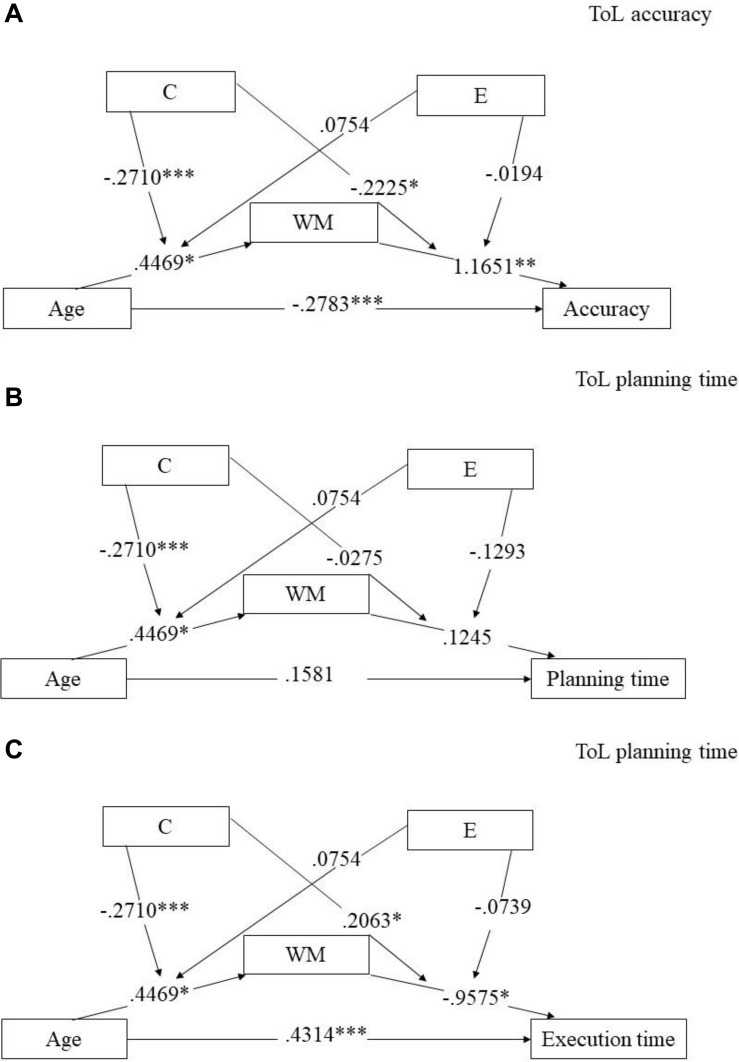
Moderated mediation analyses on ToL accuracy **(A)**, ToL planning time **(B)**, and ToL execution time **(C)** for the whole sample.

The moderated mediation results showed that the direct effect of age on ToL accuracy was significant (*b* = −0.0327, *p* = 0.0005, bootstrap SE = 0.0092, 95% bootstrap CI [−0.0508, −0.0146]). We also noted that the conditional indirect effect (mediator) of WM on the relationship between age and ToL accuracy was significantly moderated by culture and education. Results showed significant effects of WM in the Malaysian sample but not for the British sample (all CIs contained zero) (see Figure 4A). Specifically, in the Malaysian sample, the mediation effect of WM, that is the conditional indirect effect, was significant at low levels (centering on M − SD; *b* = −0.0236, bootstrap SE = 0.0080, 95% bootstrap CI [−0.0413, −0.0093]) and medium levels of education (centering on M; *b* = −0.0197, bootstrap SE = 0.0084, 95% bootstrap CI [−0.0369, −0.0040]), but not at high levels of education (centering on M + SD; *b* = −0.0161, bootstrap SE = 0.0129, 95% bootstrap CI [−0.0429, 0.0076]). This meant that the mediation effect of WM on age differences in TOL accuracy was only significant for specific levels of the culture variable (i.e., the Malaysian sample) and the education variable (i.e., low and medium levels).

Although there was no significant age by culture interaction for ToL planning and execution times, there were significant main effects of age and culture reported in ToL execution and that education was a significant covariate in ToL planning. We then explored further with similar moderated mediation analyses for both ToL planning and execution times.

Results showed that the conditional indirect effect (mediator) effect of WM on the relationship between age and ToL planning was not significantly moderated by culture and education (all CIs contained zero) (see Figure 4B). The direct effect of age on ToL planning was not significant (*b* = 0.0708, *p* = 0.0551, bootstrap SE = 0.0367, 95% bootstrap CI [−0.0016, 0.1431]). Education as a moderator was not significant as evidenced by their interaction effects in the regressions, both *p*s > 0.2478. The interaction effect for age by culture on WM was significant, *b* = −0.2549, *p* < 0.001 but not for WM by culture, *b* = −0.0552, *p* = 0.8130. These findings showed that both culture and education as moderators with WM as a mediator did not contribute to the age-related difference on ToL planning in the whole sample.

Similar to ToL planning, results showed no significant moderating effect of culture and education on the conditional indirect (mediator) effect of WM on ToL execution time (all CIs contained zero) (see Figure 4C). There was a significant direct effect of age on ToL execution time (*b* = 0.1203, *p* < 0.001, bootstrap SE = 0.0190, 95% bootstrap CI [0.0829, 0.1577]) as well as significant indirect effects (moderator) of culture by age to WM and culture by WM to ToL execution, both *p*s < 0.0254, but not for education, both *p*s > 0.4025.

These results clearly show the differences between the two cultures (British vs Malaysians), in that the age-related variance in ToL accuracy was accounted for by WM capacity for the low and medium education level only in the Malaysian sample.

## Discussion

As predicted, we found an interaction between culture (British vs Malaysian) and age in ToL accuracy, but not in planning and execution time. Our moderated mediation analysis showed that the overall effect of age on ToL accuracy was mediated by WM, and that this effect was specific to low and medium education groups in the Malaysian sample. In other words, age effects on ToL in the Malaysian sample may be driven by WM deficits, particularly in those with lower levels of education. We did not find similar effects in the British group. This fits with suggestions that tasks which may be dependent on culturally dependent skills may show diverging age trajectories in different cultures (Park et al., 1999). For the measure of speed of performance on the ToL with culture and education included as moderators, we did not find any indication that WM was a mediating factor on age relationships with planning or execution time. In our analyses, we did find that both British and Malaysian older adults were slower in executing ToL moves, but this age effect was not mediated by WM in any of our participant groups. Given that the older Malaysian sample were both slower and less accurate on the ToL task, this indicates that different attitudes towards speed/accuracy trade-offs could not explain the culture effects (in contrast to Agranovich et al., 2011).

Our findings are consistent with Unterrainer et al. (2004) in that better ToL performance was associated with shorter movement execution time. While we did not find any differences in the planning time across age and cultures, we did observe a quicker response in execution time in our young adults. Whether or not the faster or slower execution reflects confidence or eagerness in their problem-solving steps remains unanswered. However, our data showed that older adults were slower in execution times suggests that it is not entirely about confidence but rather older adults struggle with the cognitive load presented in the problems, and created some loss in accuracy specifically for the Malaysian older adults. Future research will be needed to explore these possibilities.

Why might the older Malaysian adults in our sample score more poorly on the ToL task? Previous studies indicate that tasks of WM and theory of mind show a similar pattern of interactions involving age and culture when comparing Western and Eastern samples (Hedden et al., 2002). Many previous studies indicate that a key predictor of ToL performance is WM capacity (Welsh et al., 1999; Rönnlund et al., 2001; Gilhooly et al., 2002; Asato et al., 2006; D’Antuono et al., 2017). The pattern of interaction between age and culture that we found was similar for WM and ToL accuracy: age differences were greater in the Malaysian compared to the British sample. Our results showed further that WM was a partial mediator of the relationship between age and ToL accuracy. To solve each problem effectively, participants needed to mentally work out a plan of moves, then as they went to move the discs they needed to recall their plan and amend it as needed. The cognitive load in terms of WM progressively became higher as participants completed increasingly long and more difficult trials.

Both verbal and visuospatial WM play a role in the ToL task (Phillips, 1999), and older adults may require both verbal and visuospatial WM capacity to a greater extent in order to complete the task (Phillips et al., 2003). In the current study we only assessed verbal WM, which has been argued to be important for verbally working out and rehearsing the task (Phillips, 1999). Further, having good visuospatial memory appears to also contribute to ToL success (Cheetham et al., 2012), particularly during preplanning (Gilhooly et al., 1999; Phillips, 1999).

A visuospatial strategy might entail mentally visualising the sequential movement of balls on the pegs (Cheetham et al., 2012) which may for some participants be more important than a verbal strategy (Casey et al., 1991; Kozhevnikov et al., 2005). Different individuals can be observed using quite different strategies when solving ToL trials such as pointing, gesturing, subvocal articulation, or speaking aloud. It would be interesting to code these different verbal and visual strategies in future work, to see if they are influenced by age and culture. Given that both verbal and visuospatial resources are likely to be important when completing ToL tasks, it is important that future studies look at both types of WM when looking at age and culture effects.

Other than visuospatial and WM, others have reported that fluid intelligence is a significant predictor of ToL success (Unterrainer et al., 2004; Zook et al., 2004; Köstering et al., 2014; D’Antuono et al., 2017). This evidence suggests that EF may be related to fluid intelligence (Duncan et al., 1995). Zook et al. (2006) reported that fluid intelligence is a better predictor on ToL performance compared to age, education and vocabulary. This is contrary to the findings from Bugg et al. (2006) who reported age-related variance in ToL after accounting for fluid intelligence. We did not include any fluid intelligence measures in our study, and instead had included MoCA as a general cognitive screen. Our intention for the MoCA was to exclude possible mild cognitive impairment in our sample, and our initial analyses showed a difference between the British and Malaysian older adults, although both samples average scores were above 26 and there was very limited variance. However, we were unable to collect data on some of our British older adults on the MoCA, so this is quite a small sample. In future it would be interesting to analyse whether fluid intelligence or other sensitive indicators of cognitive ability might explain culture and age effects on ToL performance.

In order to explore which factors might explain variance in ToL performance in our study, we included education and subjective SES in the subsequent ANOVA analyses as covariates. Our results showed that education was a significant contributor in ToL accuracy, ToL planning and WM but not for SES. Our moderated mediation findings showed that older Malaysians with lower education levels have performed more poorly in the ToL task. This is consistent with previous studies indicating that higher education levels contributed towards higher ToL accuracy (Boccia et al., 2017; D’Antuono et al., 2017). Having a higher education level may be indicative of having crystallized knowledge and preserved cognitive function, which could possibly help older participants in solving the problems effectively. Yet the older adults in our sample had a poorer performance in ToL despite having more years of education than young adults. One possibility is that there are qualitative differences in education since economic prosperity is higher in the United Kingdom compared to Malaysia (Roser et al., 2013), suggesting that the protective factors of higher education works differently in our sample. Similar to education, the older adults in our sample reported higher subjective SES than young adults. This contradicts previous findings in children that participants from higher SES perform better in ToL tasks (Malloy-Diniz et al., 2008; Naeem et al., 2018).

We used a physical version of ToL (wooden pegs and balls) which minimized potential technological barriers in older adults. Many of our older adult participants reported that they found the task to be enjoyable despite the numerous problems and increasing level of complexity. This indicates that participants were engaged in the task and that any performance was unlikely to be attributable to motivation or engagement. However, we note on the limited reliability of the ToL task. Some have reported low reliability for the original ToL and the improved ToL-Revised or ToL-Freiburg with more items has demonstrated higher reliability (Humes et al., 1997; Schnirman et al., 1998; Berg and Byrd, 2002; Köstering et al., 2015). They argued that the ToL version proposed by Shallice (1982) had questionable structural properties, e.g., underspecification of cognitive processes, which contributes to poor reliability and construct validity, particularly in some clinical samples.

One key limitation in our study is that our population is rather limited in terms of socio-demographic features. Our sample represents a group of individuals from relatively high socioeconomic backgrounds in similar environments (large urban cities), which may constrain effects involving SES or education. Further, the relatively high SES background is more evident in the Malaysian sample for the study was conducted in English. Although the official language in Malaysia is Malay, the medium of instruction in this study was in English. The Malaysian older adults were taught in the British Cambridge education system and opted to complete the tasks in English despite the availability to do the task in other languages (Malay, Mandarin).

Previous studies have indicated that Hong Kong samples of children show better EF performance compared to United Kingdom counterparts, but they also show that this effect is not observed for their (young or middle-aged) parents (Ellefson et al., 2017). In our data we found that ToL accuracy did not differ between Malaysia and the United Kingdom for younger participants. However, a large difference reflecting poorer performance for the Malaysians was found for older participants. While these studies differ in cultures sampled and exact tasks used, they suggest that culture effects on cognition may be very different depending on the age group sampled. Future studies covering the whole of the lifespan development, and assessing additional factors which might contribute to generational or developmental differences would be useful to better understand this phenomenon.

In conclusion, our results showed age differences such that older adults were slower and less accurate on the ToL task. There was also an interaction with culture, indicating that older Malaysians were least accurate on the task. The moderated mediation analysis indicated that the age differences in ToL performance in the Malaysian group were mediated by individual differences in WM capacity, specifically for those with lower and medium levels of education, with the mediation effect being strongest at low levels of education. This effect was not observed in the British sample. This finding indicates that the difficulties that older adults from Malaysia showed on the ToL task may be driven by a lower WM capacity, which may be linked to the participants’ educational trajectories. These findings may tentatively indicate more accelerated processes of cognitive ageing in the Malaysian sample, linked to different experiences of protective factors such as wealth, healthcare, occupational or educational experiences. Potential causes of an accelerated cognitive ageing are multiple such as physical health status and frailty. This study did not aim at exploring all of these potential factors, but future research programs testing these possibilities are needed to understand why cross-national differences in ageing are observed. Our findings indicate that the trajectory of cognitive ageing can significantly differ across countries, and therefore substantial future research efforts are needed to understand these inequalities in cognitive ageing.

## Data Availability Statement

The original contributions presented in the study are included in the article/supplementary material, further inquiries can be directed to the corresponding author/s.

## Ethics Statement

The studies involving human participants were reviewed and approved by Sunway University Research Ethics Committee (code: SUREC2018/040) and University of Aberdeen School of Psychology Ethics Committee (code: PEC/3904/2018/6). The patients/participants provided their written informed consent to participate in this study.

## Author Contributions

LP and MY were responsible for the majority of the planning, data analysis, writing of this manuscript, oversaw the project, provided advice and revisions, and were the lead investigators on the larger project from which this data was taken. LL and AS were part of the research team on the larger project and contributed through intellectual collaboration, data collection, data analysis, and manuscript revisions. CT was responsible for parts of the statistical analyses and contributed to the manuscript revisions. All authors contributed to the article and approved the submitted version.

## Conflict of Interest

The authors declare that the research was conducted in the absence of any commercial or financial relationships that could be construed as a potential conflict of interest.
